# Psychological whiplash: Factors Linked to Peritraumatic Distress in Older Adults Five Days After Missile Attacks

**DOI:** 10.1093/geroni/igaf122.4343

**Published:** 2025-12-31

**Authors:** Boaz Ben-David, Efrat Arieli Zaks, Yuval Palgi

**Affiliations:** Reichman University, Herzliya, Tel Aviv, Israel; Reichman University, Herzliya, Tel Aviv, Israel; University of Haifa, Haifa, Hefa, Israel

## Abstract

The rise of civilian-targeted warfare, including ballistic missile and drone (BMD) attacks, has globalized the need for rapid, trauma-informed mental health care for older adults. This study contributes to system-level innovation by identifying, in the immediate days following exposure, clinical predictors of peritraumatic distress (PD), a key risk factor for long-term psychopathology, in aging populations. Amid the Iran-Israel-US armed conflict, a 12-day conflict in June 2025 with daily BMD attacks affected tens of thousands of Israelis (infrastructure damage, evacuations, injuries and deaths), underscoring the importance of mental health preparedness. Yet, the psychological toll of BMD attacks on civilians has mostly been explored only months after exposure, and even less is known about their immediate impact on older adults. Two to five days after the cease-fire, we surveyed a national sample of 710 Israeli adults aged 60–85 (M = 71.86). More than half (56%) exceeded the clinical threshold of PD (PD-Inventory score ≥14). Logistic regressions (Nagelkerke-r²=.53), adjusting for sociodemographic and situational factors, revealed five significant predictors: (A) Exposure to the BMD trauma (OR = 2.3); (B) Pre-existing probable-PTSD from the October-7 attack (OR = 4.9); (C) Sleep difficulties (OR = 1.1); (D) Intensive media engagement (OR = 1.3); and (E) Decreased national identity (OR = 0.7). Findings illustrate the severe immediate psychological impact of BMD attacks on older adults. They identify scalable, clinically actionable indicators (behavioral reactions, pre-existing conditions and emotional factors) of acute stress within days of large-scale national trauma. Health systems globally can strengthen age-friendly mental health care by integrating such early screening tools into post-crisis response.

